# A Lifespan Development Theory of Insecure Attachment and Internalizing Symptoms: Integrating Meta-Analytic Evidence via a Testable Evolutionary Mis/Match Hypothesis

**DOI:** 10.3390/brainsci11091226

**Published:** 2021-09-16

**Authors:** Or Dagan, Ashley M. Groh, Sheri Madigan, Kristin Bernard

**Affiliations:** 1 Department of Psychology, Stony Brook University, Stony Brook, NY 11794, USA; kristin.bernard@stonybrook.edu; 2 Department of Psychological Sciences, University of Missouri-Columbia, Columbia, MO 65211, USA; groha@missouri.edu; 3 Department of Psychology, Alberta Children’s Hospital Research Institute, Alberta Children’s Hospital, Calgary, AB T3B 6A8, Canada; sheri.madigan@ucalgary.ca

**Keywords:** attachment, deactivating, hyperactivating, internalizing symptoms, lifespan, orientation tendency

## Abstract

Attachment scholars have long argued that insecure attachment patterns are associated with vulnerability to internalizing symptoms, such as depression and anxiety symptoms. However, accumulating evidence from the past four decades, summarized in four large meta-analyses evaluating the link between insecure attachment *subtypes* and internalizing symptoms, provide divergent evidence for this claim. This divergent evidence may be accounted for, at least in part, by the developmental period under examination. Specifically, children with histories of deactivating (i.e., insecure/avoidant) but not hyperactivating (i.e., insecure/resistant) attachment patterns in infancy and early childhood showed elevated internalizing symptoms. In contrast, adolescents and adults with hyperactivating (i.e., insecure/preoccupied) but not deactivating (i.e., insecure/dismissing) attachment classifications showed elevated internalizing symptoms. In this paper, we summarize findings from four large meta-analyses and highlight the divergent meta-analytic findings that emerge across different developmental periods. We first present several potential methodological issues that may have contributed to these divergent findings. Then, we leverage clinical, developmental, and evolutionary perspectives to propose a testable lifespan development theory of attachment and internalizing symptoms that integrates findings across meta-analyses. According to this theory, subtypes of insecure attachment patterns may be differentially linked to internalizing symptoms depending on their mis/match with the developmentally appropriate orientation tendency toward caregivers (in childhood) or away from them (i.e., toward greater independence in post-childhood). Lastly, we offer future research directions to test this theory.

## 1. Introduction

Internalizing disorders of depression and anxiety are the most prevalent diagnosed mental illnesses in children [1,2], adolescents [3,4], and adults [5,6]. Moreover, the prevalence of depression and anxiety symptoms across all age groups has been increasing over the past few decades [7,8], and they are the most disruptive disorders in terms of disease burden [9,10]. Accordingly, internalizing symptoms, their etiology, treatment, and ultimately prevention are of high public health importance [11,12]. Thus, a better understanding of which factors contribute to vulnerability to internalizing symptoms is essential for research concerning the development and persistence of these symptoms across the lifespan.

One robust characteristic and potential cause of internalizing symptoms across all developmental stages is the real or perceived loss of emotional ties to close others, which tend to increase interpersonal distress (e.g., over-dependency, worrying, and feelings of loss and abandonment [13,14,15]). This comes as no surprise, given the evolutionary adaptive benefits of maintaining close social bonds [16,17]. Strategies of managing interpersonal distress are reflected, among others, in an individual’s *attachment pattern*. An attachment pattern is commonly referred to as an emotional bond with a caregiver (and later, with close peers and/or romantic partners) that, when optimized, can provide an individual with a ‘secure base’ to rely on in times of need. Individuals who are insecurely attached, such that their ability to use the caregiver as a secure base is compromised, often have close interpersonal relationships devoid of effective soothing capacities [18] and can have difficulties regulating their emotional and psychological stress reactivity [19,20,21,22]. Compromised processing and management of interpersonal distress, in turn, is linked to internalizing symptoms across the lifespan [23,24,25]. These links between insecure attachment patterns and internalizing symptoms across the lifespan have been extensively explored in the past four decades.

In this paper, we review results from four meta-analyses that assessed the link between specific patterns of insecure attachment and internalizing symptoms in childhood, adolescence, and adulthood. We first provide an overview of how behavioral and representational attachment patterns throughout the lifespan are most commonly assessed in the developmental psychology research culture. We then review the conceptual links previously offered between attachment and internalizing symptoms and summarize the meta-analytic evidence for this link across the lifespan. Finally, we provide a critical analysis of the results. We first offer methodological explanations for the divergent findings. Then, we draw upon the developmental–evolutionary mismatch hypothesis to develop a testable theory that may advance our understanding of the association between insecure attachment patterns and internalizing symptoms across human development [26]. The theory, which we here refer to as *the lifespan developmental theory of attachment and internalizing symptoms*, predicts that one’s risk for developing internalizing symptoms in a specific developmental stage (i.e., childhood versus adolescence and adulthood) depends on the match or mismatch (hereafter, mis/match) between one’s insecure attachment pattern subtype and the developmentally appropriate orientation tendency toward or away from caregivers (i.e., avoiding proximity in childhood or pursuing greater independence in post-childhood). In the end, we propose a manner in which future research can test our proposed theory.

## 2. Attachment Assessment across the Lifespan

### 2.1. Attachment in Infancy and Early Childhood

Attachment behavior has been conceptualized as characterizing human beings “from the cradle to the grave” (Bowlby, 1969); as such, attachment patterns have been assessed throughout human development. In infancy, the gold standard assessment of attachment is an observational paradigm of infant–parent interactions: the Strange Situation Procedure (SSP [27]). The SSP is designed to activate the infant’s attachment system through brief separations from the parent; expectations of the parent’s availability are assessed by observing the infant’s behavior when reunited with the parent following brief separations [27]. Infants who are classified as securely attached are thought to have experienced repeated interactions with an emotionally available caregiver. Thus, following separations in the SSP, securely attached infants tend to quickly reestablish and maintain contact with the parent until comforted and show readiness to return to play [28].

Unlike securely attached infants, insecurely attached infants have likely experienced caregivers that are unavailable or inattentive in times of distress, leading them to develop alternative strategies to regulate their distress [29,30]. Ainsworth and colleagues [27] identified two patterns of insecure attachment, *insecure/avoidant* and *insecure/resistant*. Infants classified as insecure/avoidant tend to limit proximity seeking upon reunion with the parent; these infants tend to exhibit distant, self-reliant behaviors and expressions and direct attention away from the caregiver upon reunion [27,31]. Insecure/resistant infants tend to exhibit strong proximity-seeking behavior prior to separation from the caregiver; become increasingly distressed during separations; and upon reunion, exhibit simultaneous proximity seeking (e.g., crying or clinginess) with passivity or anger outbursts (e.g., hitting or tantrums [31,32]).

In addition to being classified as secure, insecure/avoidant, or insecure/resistant based on observed behavior in the SSP, an infant may also be classified as disorganized [33]. Attachment disorganization is thought to develop either in harsh rearing environments [34]; in the context of disrupted, frightening, or frightened parental behavior [35,36]; and/or when parents have failed to resolve past histories of trauma or grief [37]. Infants with disorganized attachment tend to exhibit fleeting disoriented and anomalous behaviors (e.g., misdirected, incomplete and interrupted movements and expressions) in the context of the SSP. These behaviors are understood as manifestations of the confusion the infant experiences between the parent as a safe haven and simultaneously as a source of threat or fear [35].

### 2.2. Attachment in Adolescence and Adulthood

By adolescence, early attachment-relevant experiences with caregivers are thought to become internalized in the form of cognitive-affective representations. In developmental psychology, attachment representations are most commonly assessed via the Adult Attachment Interview (AAI [38]). The AAI is a semi-structured interview in which individuals are asked about past experiences with their primary caregivers and how these experiences may have affected their development and personality. Attachment classifications are derived based mainly on the coherence of individuals’ discourse in the AAI [39]. That is, the AAI is coded based on an individual’s manner of speech during the interview, with higher coherence codes denoting an internally consistent but not emotionally overwrought manner of speech when describing early and current experiences and with caregivers and impacts on current functioning and relationships.

The AAI reflects discursive behavior that mirrors the behavior of the corresponding infant attachment classifications. Similar to infants who are classified as securely attached, adults who are classified as secure/autonomous in the AAI flexibly explore their environment. Whereas infants do so in the context of the physical environment, secure adults do so in the context of the ‘mental environment’, such that they are able to evaluate past and present experiences with their caregivers without becoming emotionally caught up in or cut off from these experiences. Secure narratives are thus characterized by a coherent and collaborative manner of speech [39].

Similar to insecure/avoidant infants, adults with insecure/dismissing representations based on the AAI tend to exhibit physiological reactivity suggestive of inhibition of negative emotional arousal when confronted with stressful questions (i.e., about separation and rejection [20,40]). During the AAI, the insecure/dismissing attachment pattern is predominantly indicated by the idealization of caregivers (i.e., characterization of a past relationship with caregivers in a highly positive manner with the inability to support such characterizations with autobiographical memories) and/or insistence on lack of memory of childhood events [39]. Congruent with insecure/resistant infants’ exaggeration of proximity-seeking behaviors upon separation and reunion with their caregivers during the SSP, insecure/preoccupied adolescents and adults become emotionally overwhelmed when discussing attachment relationships with one or more caregivers during the AAI. The insecure/preoccupied attachment pattern is reflected in AAI discourse via heightened expression of anger when discussing negative experiences in relation to one or more caregivers and/or difficulty in finding words and focusing on such experiences [39].

Lastly, similar to disorganized infants, disorganized adults—commonly referred to as ‘unresolved’—tend to exhibit disorientation during the discussion of past abuse or traumatic grief via lapses in monitoring of reasoning or discourse [35]. Like disorganized attachment, an unresolved attachment classification is assigned in addition to a classification of secure/autonomous, insecure/dismissing, or insecure/preoccupied.

## 3. Insecure Attachment and Internalizing Symptoms across the Lifespan: Conceptual Links

### 3.1. Infancy and Childhood

Bowlby hypothesized about links between early insecure attachment and heightened vulnerability to depression and anxiety; his framework was that “the psychology and psychopathology of emotion is […] the psychology and pathology of affectional bonds” [41] (p. 130). He proposed that anxiety is rooted in early distress regarding the availability of attachment figures [42], a hallmark of insecure attachment. Bowlby [43,44] also proposed that prolonged separation or a loss of a primary caregiver early in life, or failure to form a consistent and satisfying emotional bond with a caregiver, would lead to negative representations of the self (e.g., ‘unlovable’) and other (e.g., ‘untrustworthy’). These negative representations, in turn, are thought to enhance an experience of the relational world that is stressful, which may trigger depressive symptoms.

Following Bowlby’s propositions, a number of theorists have postulated that both insecure/avoidant and insecure/resistant attachment patterns pose a risk for childhood internalizing symptoms, given that both insecure patterns entail expectations about the unavailability of attachment figures in times of need. However, there may be different etiological pathways toward internalizing symptom vulnerability depending on the type of insecure attachment [45]. Through repeated interactions with their unresponsive attachment figures, insecure/avoidant children may learn that they are unlovable or inadequate; furthermore, they may generalize their perception of their caregivers’ inattentiveness or unavailability to perceive others as non-supportive and uncaring. These learned expectations from others may develop into behavioral and emotional tendencies that are characteristic of internalizing symptoms (e.g., inhibition of emotional arousal to prevent potential interpersonal distress, feelings of alienation and loneliness [46]). Alternately, and in line with their failures to obtain consistent availability despite repeated attempts across situations, insecure/resistant children may develop chronic concerns about the availability of the caregiver in times of need. As a result, they may experience unregulated fear and worry, rendering them vulnerable to future internalizing symptoms.

Some theorists, however, have proposed that only individuals with insecure/resistant attachment histories, but not insecure/avoidant, are more prone to developing internalizing symptoms [47,48,49]. The premise of these theories has been that children classified as insecure/resistant may have more difficulty in regulating their emotions in novel situations compared to those classified as insecure/avoidant since they are overly dependent on their caregivers and on their proximity at times of need. As a result of these frequent acute experiences of emotion dysregulation, it was theorized that insecure/resistant children may have self-perceptions of weakness and helplessness, themselves characteristics of internalizing symptoms. Relatedly, the extensive attention insecure/resistant children pay to future threats inevitably engages them in ‘painfully reliving’ these future fearful experiences [50].

Limited theorizing has underlined the potential link between attachment disorganization and internalizing symptoms. The common hypothesis has been that disorganized children’s contradictory or fragmentary behaviors observed during the SSP may indicate a breakdown of emotion regulation strategies when in distress [51]. As such, disorganized children experience fear and helplessness when encountering emotionally overwhelming situations, rendering them highly vulnerable to symptoms of both depression and anxiety [52,53].

### 3.2. Adolescence and Adulthood

Unlike the various theories regarding differential associations between insecure attachment subtypes and internalizing symptoms in childhood, the ‘conventional wisdom’ has been that, in adolescence and adulthood, insecure/preoccupied individuals are more vulnerable to internalizing symptoms than insecure/dismissing individuals [54]. Insecure/preoccupied individuals exhibit a pattern of conversational discourse in the AAI that has been thought to reflect attentional fixation on painful attachment-related memories [39,55]. This pattern of excessive attention to such painful memories, and hence to an underlying chronic distress, may enhance these individuals’ proneness to experience internalizing symptoms [56,57]. In contrast, insecure/dismissing individuals use an avoidance strategy, which serves to minimize distress and suppress negative emotion related to past attachment-related experiences [20,58]; such distress minimization, in turn, potentially reduces their vulnerability to internalizing symptoms. Although unresolved attachment representations are overrepresented in clinical samples, and thus considered a potent indicator of risk for later psychopathology [59], theorizing on the links between unresolved attachment and internalizing symptoms is currently underdeveloped.

## 4. Insecure Attachment Subtypes and Internalizing Symptoms across the Lifespan: Empirical Findings

The rich theoretical conceptualizations of the link between insecure attachment patterns and internalizing symptoms across the lifespan, described above, have been extensively tested across all age groups. However, empirical findings regarding these associations have varied across developmental age periods (i.e., childhood, adolescence, and adulthood) in terms of whether avoidant/dismissing or resistant/preoccupied attachment relate to internalizing symptoms.

### 4.1. Childhood

Two meta-analyses have examined the link between attachment patterns evaluated via observational assessments and internalizing symptoms in childhood [53,60]. The findings from both meta-analyses found support for a link between insecure attachment in early life and internalizing symptoms (*d* = 0.15, 95% CI (0.06, 0.25); [60]; and *d* = 0.19, 95% CI (0.09, 0.29); [53]). These findings indicate that insecurity (vs. security) in early childhood is weakly associated with internalizing symptoms.

Breaking down the findings into subtypes of insecure attachment (see Table 1), children with insecure/avoidant attachment had higher internalizing symptoms than both secure children (*d* = 0.29, 95% CI (0.12, 0.45); [53]) and children of all other attachment patterns combined (*d* = 0.17, 95% CI (0.03, 0.31); [60]). In contrast, insecure/resistant children did *not* show a difference in internalizing symptoms compared to secure children (*d* = 0.10, 95% CI (−0.12, 0.32); [53]) or children of all other attachment patterns combined (*d* = 0.03, 95% CI (−0.11, 0.17); [60]). Of note, although the direction of the meta-analytic association indicated lower internalizing symptoms in children who were classified as insecure/resistant than those classified as insecure/avoidant, this difference was not statistically significant (*d* = −0.17, 95% CI (−0.41, 0.06); [53]). Lastly, disorganized children did not differ in internalizing symptoms from securely attached children (after controlling for publication bias; *d* = 0.09, 95% CI (−0.02, 0.23); [53]) or children of all other non-disorganized attachment patterns combined (*d* = 0.08, 95% CI (−0.06, 0.22); [60]).

### 4.2. Adolescence and Adulthood

Adolescents and adults who were classified as insecurely attached in the AAI had higher levels of depressive symptoms (*d* = 0.21, 95% CI (0.08, 0.33); [61]), though not anxiety symptoms (*d* = 0.07, 95% CI (−0.02, 0.15); [62]), than those who were classified as securely attached. When breaking down insecure attachment pattern subtypes, the patterns of associations between insecure subtypes and internalizing symptoms in the adolescent and adult samples are *in direct contrast* to the results of the childhood meta-analyses (see Table 1). Insecure/dismissing adolescents and adults did not differ from their securely attached counterparts in either depressive or anxiety symptoms (*d* = 0.09, 95% CI (−0.03, −0.22) and *d* = −0.02, 95% CI (−0.10, 0.05), respectively). In contrast, individuals who were classified as insecure/preoccupied had significantly more depressive and anxiety symptoms than securely attached individuals (*d* = 0.48, 95% CI (0.30, 0.65) and *d* = 0.35, 95% CI (0.19, 0.50), respectively). When comparing the two insecure groups with one another, insecure/preoccupied individuals had higher depressive and anxiety symptoms than insecure/dismissing individuals (*d* = 0.34, 95% CI (0.19, 0.50) and *d* = 0.31, 95% CI (0.15, 0.47), respectively).

Lastly, unresolved adolescents and adults endorsed more depression and anxiety symptoms compared to non-disorganized individuals (*d* = 0.29, 95% CI (0.14–0.44) and d = −0.29, 95% CI (0.16, 0.42), respectively). However, this pattern of results for anxiety symptoms was driven by the secondary classification of preoccupied (i.e., disorganized/preoccupied representation).

## 5. An Overview of Results across the Lifespan

Considering the meta-analyses conducted on attachment in early childhood [53,60] and in adolescence and adulthood [61,62], a complex and somewhat unexpected picture comes to light. A deactivating attachment strategy in the early life course (i.e., insecure/avoidant in the SSP) is associated with increased concurrent and future internalizing symptoms, but a deactivating strategy in adulthood (i.e., insecure/dismissing in response to the AAI) is not. In contrast, a hyperactivating attachment strategy in the early life course (i.e., insecure/resistant in the SSP) is not linked to internalizing symptoms, whereas a hyperactivating strategy in adolescence and adulthood (i.e., insecure/preoccupied in response to the AAI) is linked with heightened internalizing symptoms.

Divergent links between insecure attachment patterns and internalizing symptoms in early versus late developmental stages may have multiple, non-mutually exclusive explanations. First, we consider how discrepancies may occur due to methodological issues, such as different informants for internalizing symptoms, variability in the phenomenology of internalizing symptoms across different developmental periods, and issues with the measurement of attachment. Second, we propose that discrepancies may be due to the mis/match between each insecure attachment type (deactivating versus hyperactivating) and the developmental stage in which the links with internalizing symptoms are measured (childhood versus adolescence/adulthood). We elaborate on these potential possibilities below.

## 6. Methodological Considerations Regarding Assessing Internalizing Symptoms and Attachment across the Lifespan

To further understand the divergent links between attachment patterns and internalizing symptoms across the lifespan, we first consider methodological issues that pertain to assessing both constructs across different developmental stages. We highlight three age-specific measurement considerations that might have contributed to these divergent results: variability across age in the informant when assessing internalizing symptoms; changes in how internalizing symptoms are expressed across development; and differences in the measurement of attachment patterns across development.

### 6.1. Different Informants for Assessments of Internalizing Symptoms

The assessment of internalizing symptoms often relies on different informants in childhood compared to adolescence and adulthood. Specifically, in the vast majority of the childhood studies included in the meta-analyses by Groh et al. [60] and Madigan et al. [53], children’s internalizing symptoms were rated by parents or teachers or by trained clinicians or observers. In contrast, most of the studies included in the meta-analyses of adolescents and adults used self-report measures of depression and anxiety symptoms.

Different informants may contribute to the divergent findings regarding the link between attachment and internalizing symptoms across development. In childhood, internalizing symptoms may be harder to observe and accurately diagnose due to their more covert nature [63]. Children’s internalizing symptoms may not come to adults’ attention because they may not be as disruptive as other more overt mental health or behavioral symptoms (e.g., externalizing behaviors). This, in turn, raises a potential issue with the validity of third-party observers’ ratings of internalizing symptoms in childhood. Relatedly, young children’s relatively limited verbal and metacognitive skills compared to those of adults may hinder their capacity to describe internal feeling states and explicitly express internalizing symptoms [64,65], making these symptoms at this early developmental period harder to observe.

Post-childhood, the reliance on self-report measures may result in under- or over-reporting in ways consistent with one’s relational patterns. For example, consistent with a deactivating attachment strategy, individuals classified as insecure/dismissing may under-report internalizing symptoms as a result of their overall tendency to minimize or deny negative experiences [54]. Similarly, the hyperactivating attachment strategy used by insecure/preoccupied individuals may contribute to an over (self)-reporting of distress and internalizing symptoms, most likely as a relational strategy to maximize one’s attachment needs (e.g., a ‘cry for help’ [66]). In line with the hypothesis that attachment patterns may be influencing self-reported symptoms, Madigan and colleagues [67] showed that comparing attachment patterns that predict self-reported internalizing symptoms in childhood yielded significantly larger effect sizes compared to when such comparisons predicted symptoms reported by third-party observers (i.e., teachers and parents).

In sum, informant bias may interfere with others detecting internalizing symptoms in childhood and may interfere with one’s own accuracy in reporting the quantity and severity of symptoms in adolescence and adulthood. Accordingly, integrating more objective ways to assess depression and anxiety across the lifespan in future research, such as clinical interviews or laboratory performance-based measures (e.g., social interaction and information processing tasks; [68,69]), might provide further support for, or challenge, the results obtained in the meta-analyses discussed here.

### 6.2. Different Phenomenology of Internalizing Symptoms

Internalizing symptoms in childhood are often harder to diagnose than in adulthood, leading to a tendency to overlook or misinterpret these symptoms in early life developmental stages [70,71]. The presentation of internalizing symptoms in childhood is affected by children’s physical, emotional, and cognitive capacities [72]. These typically underdeveloped capacities in children may result in different expressions of internalizing symptoms, which, if assessed by non-trained adult observers (such as parents and teachers, as is often the case), may lead to their inaccurate assessment in children [73]. For example, children with depression exhibit significantly higher rates of mood lability, irritability, somatic complaints (e.g., stomach pain, headaches), low frustration tolerance, and temper tantrums compared to depressed adults [74]. These symptoms, in turn, may easily be confused by the non-trained observer with multiple externalizing behaviors [46].

Taken together, it is possible that the divergent links between internalizing symptoms and attachment patterns across the lifespan may actually reflect a different group of symptoms within the same overall psychopathological factor in children and post-childhood years (e.g., internalizing disorders factor or one general psychopathology factor; [75,76]). As such, divergent associations between internalizing symptoms and attachment patterns in early and later developmental periods may in fact indicate different manifestations of the same underlying psychopathological issue across development [77].

### 6.3. Assessment of Attachment Patterns

Research on the taxonicity and factor structure of individual differences in attachment as assessed in infancy (with the SSP) and in adolescence/adulthood (with the AAI) has provided evidence that the traditional categorical coding system, which has been used in the meta-analyses described here, may subtly misrepresent the latent structure of attachment [21,78,79] (but see [80,81]). Specifically, findings from this research indicate that variation in attachment in infancy and adulthood is best captured by two weakly correlated dimensions of deactivation (i.e., avoidance in infancy; dismissing in adulthood) and hyperactivation (i.e., resistance in infancy; preoccupation in adulthood). Accordingly, attachment security, unlike the categorical model used in the meta-analyses described here, may not be a stand-alone category but rather is captured by low deactivation and hyperactivation levels. Moreover, deactivation and hyperactivation may not be mutually exclusive; instead, individuals who show deactivating behavior may also show hyperactivating behavior and vice versa. Furthermore, disorganization in infancy and unresolved attachment in adulthood load onto the hyperactivation factor (this is also supported by the meta-analytic evidence discussed in this paper, indicating that disorganized and insecure/resistant attachments are not associated with internalizing symptoms in childhood but that both are in adolescence and adulthood). Findings also indicated that the distributional properties of individual differences in attachment are more consistent with a dimensional than categorical model [78,82].

Taken together, such evidence has important implications for research on the developmental significance of attachment. For example, analyses leveraging categorical data rely on a measurement model that assumes that individuals exhibiting deactivating strategies cannot simultaneously exhibit hyperactivating strategies. Hence, the variation in levels of insecure/resistance (in infancy) and preoccupation (in adolescence and adulthood) is decreased, which is especially problematic given the low base rates of these attachment patterns. Moreover, applying a categorical structure to individual differences that vary continuously can compromise the power to detect significant links between attachment and internalizing symptoms [83].

Lastly, when thinking about assessing attachment throughout the lifespan, one should consider that attachment patterns in early life have been, more often than not, assessed only with mothers. The prioritization of mothers as the primary attachment figure in research may be relevant to better understand the link between insecure attachment patterns and internalizing symptoms in childhood. Given that infants simultaneously and independently develop attachment patterns with both mothers and fathers [84], it is possible that the link between attachment and internalizing symptoms may in fact be more accurately estimated via the assessment of an infant’s network of attachment to *both* parents instead of their attachment to only one parent (usually the mother [84,85]). Likewise, it is possible that experiences with new attachment figures in later stages of life (e.g., romantic partners) lead to changes in attachment-related expectations. Thus, post-childhood attachment patterns that are developed based on interactions with new close others may be different than the attachment patterns developed with primary caregivers that are classified from the AAI [86,87]. This additional (or shift in an overarching) attachment pattern may complicate the links between attachment and internalizing symptoms in adolescence and adulthood.

Overall, future research will benefit from increased attention to the latent structure of individual differences in attachment and will ideally assess attachment patterns with multiple parental and non-parental caregivers who constitute the child’s attachment network structure. These practices will better represent the factor and taxometric structure of attachment and will improve its ecological validity. Such methodological practices will enhance the statistical power and increase our confidence in or adjust the meta-analytic results obtained thus far.

## 7. Intermediate Summary

The methodological considerations regarding internalizing symptoms and attachment assessment across the lifespan discussed above are by no means negligible. However, a complex phenomenon such as divergent links between attachment patterns and internalizing symptoms across multiple developmental stages also requires a new theoretical framework. Integrating all the variables of interest into a cohesive theory can open new perspectives on the discrepant results obtained in the meta-analyses described here and create a basis of developing testable research questions. Relatedly, an overarching theoretical account of the meta-analytic results allows for the necessary engagement of other research disciplines outside of attachment research (such as recent evolutionary theory and research, as discussed below); this, in turn, may open the question at hand to more rigorous testing by interdisciplinary methodologies.

## 8. A Lifespan Development Theory of Insecure Attachment and Internalizing Symptoms

Despite a growing literature on attachment and internalizing symptoms, an overarching, empirically based, and testable theory for the differential associations between the subtypes of insecure attachment patterns and internalizing symptoms *across the lifespan* has yet to be formulated. Moreover, it was recently proposed that given that attachment theory is firmly rooted in evolutionary theory, a rethinking of the components of attachment theory in the context of modern evolutionary framework is needed [26,88,89]. Future research will thus be most successful in clarifying and integrating the inconsistent findings presented here if, alongside methodological considerations, it is guided by conceptual evolutionary interpretations.

From an ontogenetic evolutionary perspective, behavioral adaptations early in life, including insecure attachment patterns [88], develop as a result of an ecological pressure to increase chances for survival during the most perilous years of development and ultimately to increase reproductive fitness later in life. Some of these adaptations reflect a learning process that individuals go through via repeated interactions with their primary caregivers, whose behaviors toward their offspring provide cues about the nature of the current and potentially future environmental conditions (e.g., how dangerous these environments are and how trustworthy caretakers may be [90,91]). Although such local adaptations are thought to increase survival rates and reproductive success, these early adaptations may have unintended mental health consequences [92]. Importantly, these consequences may differ depending on the ecological context or the fit between the individual’s behavioral adaptation to the immediate caregiving environment and wider social/environmental demands of different developmental stages across the lifespan [93]. The fit between the individual’s behavioral adaptation to the immediate caregiving environment and the wider social environment may change as a result of an inherent human life course transition; that is, a transition from predominantly parental caregiving during childhood years to increased independence from them during adolescence and adulthood, which oftentimes entails various degrees of reliance on oneself and/or non-parental (e.g., peers and romantic partners) caregiving. The fit between the early adaptive relational patterns and the social/environmental demands that are present in childhood versus post-childhood years may be viewed through the lenses of a developmental–evolutionary theory: the *developmental mismatch hypothesis*.

The developmental mismatch hypothesis [93,94] assumes that mental/physical health and disease are dependent on the degree of mis/match between the affective, cognitive, and behavioral adaptations one develops in the early environment and the environment one inhabits as an adult. This hypothesis is especially intriguing in the context of the divergent meta-analytic findings on the links between attachment patterns and internalizing symptoms across the lifespan for two reasons. First, the developmental mismatch hypothesis highlights the crucial role that stability and change in one’s social/developmental environment play in the individual’s mental health [95,96]. Second, the developmental mismatch hypothesis predicts that mismatch in *either direction*—that is, maturing from a harsh environment to a more favorable one, as well as from a normative (e.g., non-excessively stressful) developmental environment into a difficult (e.g., interpersonally taxing) one—may expose individuals to mental health risks [93]. Together, these two essential tenets of the developmental mismatch hypothesis may explain how the *same* attachment strategies that are expressed in *different* social environments across human development may be associated with *varying* degrees of vulnerability to internalizing symptoms.

In the following section, we use the developmental mismatch hypothesis framework to propose that deactivating and hyperactivating attachment patterns may be differentially associated with internalizing symptoms depending on the developmental period in which they are assessed. We argue that the adaptiveness of deactivating and hyperactivating attachment patterns across the lifespan varies depending on the developmental period, thereby leading to heightened or decreased susceptibility to internalizing symptoms. Specifically, we consider the mis/match between (a) the insecure attachment pattern subtype and (b) the appropriate orientation tendency with respect to caregivers within each developmental phase (i.e., toward caregivers in childhood and away from caregivers in adolescence and adulthood; see Table 2).

### 8.1. Developmentally Appropriate Orientation Tendencies toward or away from Caregivers across the Lifespan

Maintenance of proximity to caregivers early in life—especially in humans, who are born significantly underdeveloped compared to other species [97]—has been favored by natural selection. As such, proximity maintenance to parental caregivers constitutes a fundamental feature of attachment bonds [89]. From an attachment perspective, a developmentally appropriate orientation tendency at times of need during early life is thus one that entails proximity seeking to (i.e., *toward*) parental caregivers. When experiencing distress, such an orientation allows children to use parental caregivers as a ‘secure base’ and, as such, can effectively soothe the child’s physical or emotional pain [43,98].

When parents consistently respond to children’s cues of distress, children are likely to trust in the effectiveness of their support-soliciting efforts and the availability of caregiving and effective soothing [99]. However, distrust in the availability of caregiving support at times of need may lead to children developing a tendency to orientate themselves away from parental support in order to lessen an anticipated rejection and emotional pain that are associated with parental unavailability in the short run. Such a tendency has a tradeoff: it may lead to prolonged and more frequent experiences of distress due to children’s limited capacity for emotion regulation, which may thus enhance their vulnerability to internalizing symptoms [100,101].

Adolescence is characterized by a normative transition from psychological dependence on parental figures to more emotional self-sufficiency in the service of finding one’s place in the social world of their peers [102,103]. In line with such normative transition, the functions of attachment bonds to one’s parents gradually shift toward peers and romantic partners during adolescence and adulthood [104]. Accordingly, a developmentally appropriate behavior in adolescence is oriented *away from* parental caregivers (i.e., toward increased independence from them) in the overall effort to achieve greater affective, cognitive, and behavioral autonomy [105,106]. Orienting oneself away from parental support inherently allows for a gradual formation of non-parental close relationships with peers and romantic partners, who take an increasingly larger role in providing support for attachment needs [107,108].

Given that the role of the parent as a secure base is not severed when entering adolescence and throughout adulthood, exercising orientation away from caregivers and toward greater independence from them in a healthy and adaptive manner during this transition entails an ability to verbally communicate disagreements with parents as well as ask for help when needed [109,110,111]. However, the inability to increasingly orient oneself away from caregivers (and toward greater independence) may impede successfully establishing healthy levels of self-reliance and secure non-parental adult–adult relationships [112]. Post-childhood, lacking the capacity for regulating one’s own emotion and perceiving oneself as socially isolated due to a scarcity of satisfactory peer or partner relationships increase the likelihood of experiencing internalizing symptoms [113,114,115,116]. Coupled with changes in adolescents’ psychophysiological stress reactivity [117,118], perceived social isolation may consequently exacerbate the vulnerability to internalizing symptoms in adolescence [119,120] and later on in adulthood [121].

In sum, different attachment patterns vary in the extent to which they entail an orientation tendency toward or away from parental caregivers at times of need during childhood and post-childhood developmental stages. As we elaborate below, although such orientation tendencies may be viewed as maladaptive from a psychopathological viewpoint (e.g., increased internalizing symptoms), they may also be understood as adaptive from an ontogenetic evolutionary perspective (e.g., maximizing proximity maintenance to caregivers when they are perceived to be unavailable [122]). Accordingly, the tendency to orient oneself toward or away from parental caregivers when distressed is not in itself “good” or “bad”; rather, exercising such orientations may increase or decrease interpersonal distress—thereby functioning as a risk factor for or a protective factor against internalizing symptoms—depending on the developmental stage.

### 8.2. Mis/Match between Attachment Patterns and Developmentally Appropriate Orientation Tendencies in Childhood versus Adolescence/Adulthood

#### 8.2.1. Deactivating Attachment Strategies

Deactivating attachment strategies, which are thought to develop in response to inattentive or unresponsive caregiving [30,58], are observed in the SSP in insecure/avoidant behavior and in the AAI via an insecure/dismissing discourse. Deactivating attachment strategies are characterized by the inhibition of negative emotional arousal on either the intrapersonal level (e.g., redirecting attention away from painful memories) or the interpersonal level (e.g., appearing ‘untouched’ by stressful circumstances).

In infancy and childhood, disengagement from the parental figure at times of distress—a hallmark of the insecure/avoidant behavioral pattern—may reflect a lack of confidence in the caregiver’s availability in times of need. This tendency is thought to leave children in a state of prolonged distress when confronted with interpersonal challenges due to their immature capacity for self-regulation and need for aid from parents in regulating emotions [80]. Indeed, children higher on the deactivating dimension tend to show increased electrodermal activity in response to imagined situations that involve help-seeking behaviors toward caregivers, which may reflect fear and anxiety in circumstances where parental support is expected to be unavailable [123]. Relatedly, experimentally modifying children’s interpretations of their parents’ responses to distress as supportive, coupled with training children to orient their attention to their parents when distressed, increased their trust in parental support [124,125]. Deactivating children’s lack of trust in the presence of parental support at times of need may thus be what underlines their tendency to turn *away* from parental support in times of need, opposing the developmentally appropriate orientation tendency in childhood to turn toward caregivers in such circumstances. The tendency to turn away from parental support when in need may lead deactivating children to experience more intense and prolonged distress compared to their non-deactivating counterparts, an experience that has shown to constitute a critical vulnerability factor in developing anxiety and depressive symptoms [126].

Early life interpersonal stressors, however, may *positively* influence mental health outcomes (in the form of adaptive stress responses) for some individuals in post-childhood social conditions [127]. By the time they enter adolescence, deactivating individuals may already be well accustomed to inhibiting negative emotions and avoid processing attachment-related threats at times of challenge. As Bowlby [44] suggested, deactivating the attachment system may reflect a ‘defensive exclusion’—that is, a partial or complete disengagement from processing socially stressful information by redirecting attention away from it. Such a ‘well practiced’ defensive strategy may in fact prove adaptive once one enters post-childhood years. That is, deactivation of the attachment system at times of need may be effective in buffering the encoding of negative emotional information that is increasingly present in adolescence and adulthood. This, in turn, may prevent painful experiences that result from negative interpersonal interactions [128,129], thereby decreasing the magnitude of internalizing symptoms.

Intrapersonally, insecure/dismissing individuals tend to encode and remember less threatening (but not neutral) emotional information [18,130] and exclude negative information when evaluating relationship-relevant stimuli (e.g., when presented with separation or quarrel movie scenes [131]). In addition, young adults higher on the insecure/dismissing dimension showed increased vigilance to affectively laden facial expressions and social interaction stimuli compared to their counterparts on the lower end of the insecure/dismissing dimension [132]. Such a pattern of results may be indicative of the initial, pre-conscious alertness to signs of potential rejection, which is essential in subsequently keeping such potentially hurtful material away from conscious awareness [133]. Taken together, the evidence suggests that the avoidance strategy that insecure/dismissing adolescents and young adults employ when encountering potential interpersonal threats may contribute, at least in part, to lessen the experience of attachment-related distress [129].

Deactivating attachment may prove interpersonally beneficial in two ways as individuals transition into adolescence. First, the deactivating tendency toward self-reliant distress alleviation and the minimal expression of help cues may allow individuals to engage with the developmentally appropriate exploration of the social environment of peers without being impeded by excessively relying on parental support when doing so. In this sense, deactivating attachment strategies may ease the otherwise often difficult adolescence and young adulthood transition from parental support to peer and romantic partner engagement. Second, as deactivating strategies are associated with reduced support-seeking behavior, individuals may be less likely to experience rejection by peers, experiences that are otherwise prevalent in post-pubertal developmental stages [103,115]. Overall, given that deactivating strategies are well aligned with the developmentally appropriate orientation toward greater independence from primary caregivers (and toward potential other non-parental attachment figures), adolescents and adults may experience reduced interpersonal distress, which then decreases their vulnerability to internalizing symptoms [134,135].

Of note, given that romantic partners are thought of as attachment figures [136], one may suspect that the tendency of deactivating adolescents and adults to orient themselves away from (non-parental) attachment figures at times of need will undermine their ability to establish healthy romantic relationships, which in turn may lead to increased internalizing symptoms. Two points are worth mentioning in this regard. First, evidence regarding the quality of individuals’ romantic relationships according to variation in attachment insecurity has been inconsistent. If anything, hyperactivating (but *not* deactivating) attachment in adolescence has been shown to longitudinally predict low overall satisfaction and stability in adult romantic relationships [137,138]. The ability to “let go” of parental support may in fact position deactivating adolescents to forge new relationships—even if not profound—with romantic partners who can heed the call of meeting their previously unmet emotional needs [43,139].

Second, it may be that deactivating individuals experience relatively little internalizing symptoms despite non-optimal romantic relationships. This may be the case if deactivating individuals effectively use their self-reliant emotion regulatory capacity in the context of relationship stressors to avoid escalating, if not deescalating, them altogether. Indeed, minimization of distress communication—a hallmark of deactivating adolescents and adults—tends to result in low levels of distress, at least in the short term [140,141] (but see [142]). In addition, although the romantic relationships of deactivating individuals may be less satisfying due to a stronger tendency to rely on oneself rather than the other, such relationships may also elicit less relational stressors given that the emotional investment in them is relatively low to begin with. As such, romantic relationships of deactivating adolescents and adults may confer less risk for internalizing symptoms.

In summary, deactivating children may not orient themselves toward their caregivers when they are in need, as they do not expect them to respond effectively to their signals of distress. The developmental *mismatch* between the deactivation attachment pattern (i.e., the tendency to orient oneself away from parental caregiving at times of need) and the developmentally appropriate orientation toward caregivers in childhood may thus explain why deactivating attachment strategies render children more vulnerable to be “left alone” when dealing with distress. As such, deactivating attachment in childhood may lead to heightened vulnerability to internalizing symptoms. In adolescence and beyond, however, individuals using deactivating attachment strategies may developmentally *match* the appropriate orientation tendency away—and toward greater independence—from primary attachment figures. Their independence from caregivers, albeit sometimes excessive in nature, may enable them to self-regulate interpersonal distress and potentially forge new relationships with peers and romantic partners who can fulfill previously unmet emotional needs, thereby decreasing their vulnerability to internalizing symptoms [143,144].

#### 8.2.2. Hyperactivating Attachment Strategies

Hyperactivating attachment is thought to develop in response to inconsistent availability of caregivers in times of distress [58,129]. Hyperactivating strategies are characterized by heightened expression of negative emotions, mostly anger and distress, on either the intrapersonal level (e.g., being constantly hypervigilant to threats) or the interpersonal level (e.g., excessive ‘crying for help’). This strategy is observed in the SSP in insecure/resistant behavior and in insecure/preoccupied discourse during the AAI.

Insecure/resistant children show a tendency to maximize proximity-seeking behaviors throughout the duration of the SSP, which may reflect a belief in their ability to solicit support, or at the very least obtain proximity to caregivers, if their attachment signals are strong enough [57]. Despite learning that parental support—while still available—will only be provided if excessive bids for attention are undertaken [32,53], hyperactivating children may develop a ‘hopeful’ stance that instills in them the tendency to orient themselves toward caregivers when under distress. This ‘hopeful’ stance—which may be well based on infants’ and children’s ability to make probabilistic inferences about parental responsiveness to their support signals [145]—may stand at the basis of hyperactivating children’s orientation tendency toward support soliciting from caregivers when in need. This interpersonal tendency, in turn, decreases vulnerability to psychopathology, including internalizing symptoms [100,146].

When entering adolescence and continuing to adulthood, hyperactivating individuals may face difficulties in the normative process of establishing autonomy from caregivers and continuously orient themselves toward them [102]. Unlike deactivating individuals, hyperactivating individuals tend to experience difficulties in disengaging from parents [112,147]. The emotional entanglement with their parents may cause hyperactivating individuals to have limited capacity to develop autonomy from them and establish satisfying non-familial close relationships to which they can turn to in times of need [138,148]. Moreover, although close relationships with peers and romantic partners are highly desirable to hyperactivating adolescents [149], their relational behaviors with their parents tend to generalize to non-parental relationships in ways that lead them to be perceived unfavorably by others (e.g., as “clingy”, excessively anxious, or demanding [32,149]). Relatedly, preoccupied adolescents’ excessive desire for close relationships with peers often leads to poor functioning in these relationships [149,150]. These individuals also tend to report distress, loneliness, over-dependence, and overall dissatisfaction in relationships with peers and romantic partners [138,148,151,152]. Together with such negative interpersonal experiences, the compromised capacity to disengage from parental relationships and to establish a meaningful network of close and intimate non-familial relationships that can serve as emotional support at times of need increases one’s vulnerability to both anxiety and depression symptoms [153].

Overall, hyperactivating children engage in what may be understood as a developmentally appropriate orientation tendency toward their caregivers when they are in need. Such tendency is evident by hyperactivating children’s ongoing motivation to solicit support, often through excessive means. The *match* between the hyperactivation attachment pattern and the developmentally appropriate orientation tendency toward their caregivers may lead to decreased vulnerability to experiencing internalizing symptoms. However, the inability to obtain independence from parental relationships when entering post-childhood years constitutes a developmental *mismatch* between the hyperactivating attachment patterns and the appropriate orientation tendency away (i.e., toward greater independence) from parental caregivers. Thus, hyperactivating individuals may struggle to establish independence from parents and appropriately integrate into a social environment that allows for effective soothing within meaningful non-parental attachment relationships. This inability to exercise a healthy transition into adulthood may significantly contribute to a heightened vulnerability to developing depressive and anxiety symptoms [154,155].

### 8.3. Testing the Mis/Match Hypothesis in the Context of Insecure Attachment and Internalizing Symptoms across the Lifespan

Testing whether the mis/match between insecure attachment patterns and developmentally appropriate orientation tendencies toward or away from caregivers in childhood versus adolescence/adulthood explain the divergent links between attachment and internalizing symptoms across the lifespan is essential. Thus, we draw upon the two mis/match hypothesis predictions that guide multiple health and disease evolutionary–developmental models [93,94]. The first predicted trajectory is that of individuals who transition from a stressful to a non-stressful environment. In order to adapt to stressful, non-supportive environments, children develop responses that are geared toward reducing distress via detection and monitoring of danger [156,157]. When the environmental conditions become safer and less stressful, these individuals may find that their once adaptive strategies are no longer ‘useful’, as the hyper-vigilant profile becomes at odds with the new social environment that does not call for such adaptation [158]. The second and opposite predicted developmental trajectory posits that children who grow up in safe and supportive environments develop and consolidate their interpersonal strategies in a manner that may not ‘work’ if used in later stressful environments, ultimately leading to a developmental mismatch between the once learned adaptive pattern of behavior and the current social circumstances [159,160]. Both predicted trajectories assume that mismatched environmental conditions—regardless of their life course timing—will lead to increased internalizing symptomatology (for evidence supporting the developmental mismatch hypothesis in the contexts of anxiety and depression symptoms in mice, see [161]).

Consistent with the developmental mismatch hypothesis, we propose that whether a social environment is experienced as stressful, and therefore increases one’s vulnerability to internalizing symptoms, depends on the degree to which one’s insecure attachment pattern matches with the developmentally appropriate orientation tendency toward or away from caregivers. Accordingly, the transition from an environment in which parents are primarily relied on for emotional support (and thus orientation tendency *toward* them at times of need, even if excessive, is developmentally appropriate) to one in which the source of support comes primarily from non-parental caregivers (and thus orientation tendency *away* from parental caregivers at times of need is warranted) may be experienced as increasingly distressing for hyperactivating individuals and decreasingly distressing for deactivating individuals.

**Figure 1:**
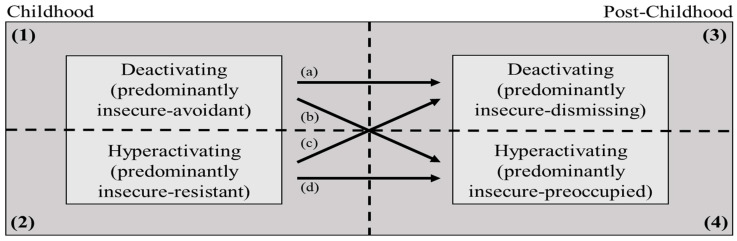
Suggested study design to test the mis/match hypothesis in the context of insecure attachment and internalizing symptoms across the lifespan. Four groups (a–d) that differ in their insecure attachment dis/continuity between childhood to post-childhood developmental periods will be assessed. Each quadrant (1–4) represents an interaction of an insecure attachment pattern and developmental period.

Specifically, children with deactivating strategies tend to orient themselves away from (i.e., avoid proximity to) parental caregivers when under distress early in life, leading to heightened experience of distress and, thus, to internalizing symptoms during childhood (Figure 1, Quadrant 1). However, deactivating strategies will prove less harmful as individuals transition to the new social environment, where the tendency to orient oneself away (i.e., toward greater independence) from parental caregiving will match the developmentally appropriate orientation tendency during adolescence and adulthood. This developmental transition from childhood to post-childhood may result in reduced distress, thereby decreasing deactivating individuals’ likelihood of developing internalizing symptoms (Figure 1, Quadrant 3).

In contrast, hyperactivating strategies constitute a match with the developmentally appropriate orientation tendency toward caregivers in early life. Although such a match does not eliminate distress, the orientation tendency toward parental support, even if excessive in nature, may result in a lower quantity and/or frequency of stressful experiences in hyperactivating children compared to deactivating children. Thus, hyperactivating children may be at a relatively low risk of internalizing symptoms (Figure 1, Quadrant 2). However, these strategies will fail to serve the purpose of distress alleviation later in life, as they are at odds (i.e., mismatched) with the post-childhood developmentally appropriate orientation tendency away (i.e., toward greater independence) from parental caregivers and toward more effective support soliciting from peers and romantic partners. Such a developmental mismatch may exacerbate experiences of interpersonal distress and, thus, may increase the likelihood of internalizing symptoms (Figure 1, Quadrant 4).

According to the mis/match hypothesis that stand at the heart of our proposed lifespan development theory of insecure attachment and internalizing symptoms, individuals who switch from one insecure pattern to another will show stability of internalizing symptoms between early and later life periods. These individuals may be less at risk for poor adjustment to both early and later social/environmental demands (as in the case of individuals switching from hyperactivating to deactivating strategies) or poorly adjusted during both developmental periods (as in the case of individuals switching from deactivating to hyperactivating strategies).

Based on the models proposed to test the development of depression in an evolutionary-based mis/match framework [94,162], future research will benefit from testing our lifespan developmental theory of attachment and internalizing symptoms by longitudinally following individuals and assessing their attachment patterns from pre- to post-childhood developmental stages. The given evidence suggests that the latent structure of attachment in infancy and adulthood is better captured by two modestly associated dimensions (rather than three or four distinct categories; [163]); thus, to maximize power, we propose assessing attachment patterns dimensionally to represent variations in individuals’ deactivation and hyperactivation attachment indices.

The proposed lifespan developmental theory of attachment and internalizing symptoms would be corroborated if the following four hypotheses were supported (see Table 3): (a) individuals who are predominantly deactivating in infancy and adulthood will show a *decrease* in internalizing symptoms from childhood to post-childhood; (b and c) individuals whose predominant insecure attachment strategies change along with the developmental transition from childhood to adolescence will exhibit stability in internalizing symptoms across development; and (d) individuals who are predominantly hyperactivating throughout development will exhibit an *increase* in internalizing symptoms from childhood to post-childhood.

## 9. Future Research

To better understand the role of insecure attachment patterns in the onset, maintenance, and potential reduction of internalizing symptoms across the lifespan, and to examine the lifespan developmental theory proposed here, existing longitudinal datasets and new prospective longitudinal study designs may prove useful. Available datasets that have assessed attachment patterns and internalizing symptoms across the lifespan (e.g., the NICHD Study of Early Child Care and Youth Development), and which may control for environmental influences across development, may well put to test the four mis/match hypotheses detailed in Table 3.

Clearly, large prospective studies are also needed. Such study designs should test the mechanisms of change proposed by the lifespan developmental theory of attachment and internalizing in the form of orientation tendencies toward/away from parental caregivers during different development stages. Attachment research has already developed assessments of behaviors that tap one’s orientation tendency toward/away from caregivers at times of need. For example, cognitive assessments of trust, such as tasks assessing levels of attentional bias toward mothers when experiencing distress, can function as indicators of orientation tendencies toward/away from caregivers (for a review of cognitive assessments of this kind, see [164]). Post-childhood, the successful shifting from reliance on parental figures to orientation tendency away from them may well be reflected in individuals’ tendency for *autonomy* from caregivers. Cognitive, emotional, and behavioral assessments of individual differences in autonomy, including self-report questionnaires by multiple informants, diary reports, and observational coding of adolescent–parent interactions (for a review, see [149]), may prove useful in indicating orientation tendencies toward parental caregiving or away (i.e., toward greater independence) from them in post-childhood years. To complement the psychological factors that drive individuals’ orientation tendencies with respect to their caregivers at times of need across the lifespan, both environmental and individual risk factors that have been shown to affect the onset or exacerbate psychopathological symptoms across developmental stages should be assessed and controlled for in analyses. Such factors may include low household income and maternal depression [165], genetic factors [166], and experiences of traumatic events [167].

Of note, the methodological limitations listed above should be addressed in future prospective longitudinal endeavors. We recommend using multiple methods in each developmental period to assess both internalizing symptoms (for recent developments in symptom harmonization methods to establish longitudinal measurement invariance across the lifespan, see [168]) and insecure attachment patterns. Such methodological practices would ensure measurement invariance and, thus, determine the degree to which the mis/match between the developmentally appropriate orientation tendency toward one’s parental caregivers and the attachment strategy in different development stages is indeed the driving force in the divergent results we highlight in this paper.

## 10. Conclusions

Examining the role that attachment plays in exacerbating vulnerability to psychopathology has not been an easy task for developmental scientists. Although more than four decades of research have resulted in substantial literature linking attachment patterns to internalizing symptoms across the lifespan, methodological and conceptual issues have posed significant challenges to summarizing this literature from a lifespan perspective. As presented here, divergent meta-analytic findings highlight the complexity of the dynamics between insecure attachment and internalizing symptoms across the lifespan. At the very least, the divergent results presented here highlight a need to evaluate and better understand the link between insecure attachment patterns and internalizing symptoms across multiple developmental periods rather than limit each study to a specific developmental stage. Assessing our testable theory laid out in this paper is the first step in this direction.

As previously noted, a crucial value of meta-analytic results is not merely an end point for a research endeavor but rather a starting point for generating more informed hypotheses [169]. In line with this proposition, we leveraged results from four meta-analyses spanning nearly four decades of research to generate a new hypothesis for future empirical studies to confirm or falsify. Of course, carrying out the task of validating the evolutionary-based mis/match hypothesis offered here is most certainly difficult and not without errors to be corrected as any programmatic research moves forward [170]. The need for empirical support on multiple levels of analyses (e.g., cognitive and emotional assessments) and potentially for cross-disciplinary validation (e.g., psychological and physiological evidence [171]) makes the assessment of the theory proposed here no different. We hope that future investigations that aim to enhance our understanding of the varied trajectories that insecure attachment patterns may take to enhance or reduce vulnerability to internalizing symptoms across the lifespan will use this theory as a launch pad from which to embark on such an empirical task.

## Figures and Tables

**Table 1:** List of meta-analyses assessing the magnitude of the links between insecure attachment patterns and internalizing symptoms.

Author (Year)	Attachment Measures	Most Prevalent Symptoms Measures	Attachment Group Comparison	*k*	N	Cohen’s *d* (95% CI)	Heterogeneity
CHILDHOOD							
Groh et al. (2012) [60]	SSP	CBCL	**A** versus B+C+D	22	3119	0.17 (0.03, 0.31)	Q(21) = 32.82 *
			C versus B+A+D	21	3078	0.03 (−0.11, 0.17)	Q(20) = 26.05
Madigan et al. (2013) [53]	SSP	CBCL, TRF	**A** versus B	21	1852	0.29 (0.12, 0.45)	Q(20) = 33.78 *
			C versus B	21	1823	0.10 (−0.12, 0.32)	Q(20) = 30.11 *
			**A** versus C	19	664	−0.17 (−0.41, 0.06)	Q(18) = 48.35 **
ADOLESCENCE AND ADULTHOOD							
Dagan et al. (2018) [61]	AAI	BDI, CES-D	Ds versus F	43	2881	0.09 (−0.03, 0.22)	Q(42) = 90.68 ***
			**E** versus F	38	2079	0.48 (0.30, 0.65)	Q(37) = 71.90 ***
			**E** versus Ds	37	1285	0.34 (0.19, 0.50)	Q(36) = 47.65
Dagan et al. (2020) [62]	AAI	BSI, SCL-90-R	Ds versus F	50	4376	−0.02 (−0.10, 0.05)	Q(49) = 68.09 *
			**E** versus F	42	3271	0.35 (0.19, 0.50)	Q(41) = 99.53 ***
			**E** versus Ds	41	2184	0.31 (0.15, 0.47)	Q(40) = 88.99 ***

Bolded letters represent the attachment classification groups associated with significantly more reported symptoms compared with the other group(s). *k* = Number of studies; N = Number of participants; AAI = Adult Attachment Interview; SSP = Strange Situation Procedure; BDI = Beck Depression Inventory (self-report); BSI = Brief Symptom Inventory (self-report); CBCL = Child Behavior Checklist (parent report); CES-D = Center for Epidemiologic Studies Depression Scale (self-report); SCL-90-R = Symptom Checklist-90-Revised (self-report); TRF = Teacher’s Report Form; A = Insecure-Avoidant; B = Secure; C = Insecure-Resistant; Ds = Insecure-Dismissing; E = Insecure-Preoccupied; F = Secure-Autonomous. * *p* < 0.05. ** *p* < 0.01 *** *p* < 0.001.

**Table 2:** The mis/match between insecure attachment strategies and the orientation tendencies toward or away from caregivers by developmental stage.

	Dominant Support Figures	Appropriate Orientation Tendency	Hyperactivating Strategies		Deactivating Strategies	
**Childhood**	Parental figures	*Toward* parental caregivers	**Match**	(Excessive) orientation tendency toward parental caregivers	**Mismatch**	Avoiding proximity to parental caregivers
**Post-Childhood**	Non-parental figures	*Away* (i.e., toward greater independence) from parental caregivers	**Mismatch**	Enmeshment with parental caregivers	**Match**	(Excessive) orientation tendency away (i.e., toward greater independence) from parental caregivers

**Table 3:** Predictions of the mis/match hypothesis in the context of insecure attachment patterns and internalizing symptoms across the lifespan per group.

Attachment Group	Prediction
(a) Continuous deactivation	Decrease in internalizing symptoms from childhood to post-childhood
(b) Child deactivation→ Post-childhood hyperactivation	Stable high internalizing symptoms across development
(c) Child hyperactivation→ Post-childhood deactivation	Stable low internalizing symptoms across development
(d) Continuous hyperactivation	Increase in internalizing symptoms from childhood to post-childhood

Each group’s letter corresponds with the trajectory letter in Figure 1.
