# Correction: Dagan et al. A Lifespan Development Theory of Insecure Attachment and Internalizing Symptoms: Integrating Meta-Analytic Evidence via a Testable Evolutionary Mis/Match Hypothesis. *Brain Sci.* 2021, *11*, 1226

**DOI:** 10.3390/brainsci12070820

**Published:** 2022-06-24

**Authors:** Or Dagan, Ashley M. Groh, Sheri Madigan, Kristin Bernard

**Affiliations:** 1 Department of Psychology, Stony Brook University, Stony Brook, NY 11794, USA; kristin.bernard@stonybrook.edu; 2 Department of Psychological Sciences, University of Missouri-Columbia, Columbia, MO 65211, USA; groha@missouri.edu; 3 Department of Psychology, Alberta Children’s Hospital Research Institute, Alberta Children’s Hospital, Calgary, AB T3B 6A8, Canada; sheri.madigan@ucalgary.ca

## Error in Table

In the original article [1], there was a mistake in Table 1 as published. The confidence interval reported for the comparison between insecure-dismissing and secure attachment adolescence and adulthood classification groups was wrong. The corrected Table 1 appears below. The authors apologize for any inconvenience caused and state that the scientific conclusions are unaffected. The original article has been updated.

## Figures and Tables

**Table 1:** List of meta-analyses assessing the magnitude of the links between insecure attachment patterns and internalizing symptoms.

Author (Year)	Attachment Measures	Most Prevalent Symptoms Measures	Attachment Group Comparison	*k*	N	Cohen’s *d* (95% CI)	Heterogeneity
CHILDHOOD							
Groh et al. (2012) [60]	SSP	CBCL	**A** versus B+C+D	22	3119	0.17 (0.03, 0.31)	Q(21) = 32.82 *
			C versus B+A+D	21	3078	0.03 (−0.11, 0.17)	Q(20) = 26.05
Madigan et al. (2013) [53]	SSP	CBCL, TRF	**A** versus B	21	1852	0.29 (0.12, 0.45)	Q(20) = 33.78 *
			C versus B	21	1823	0.10 (−0.12, 0.32)	Q(20) = 30.11 *
			**A** versus C	19	664	−0.17 (−0.41, 0.06)	Q(18) = 48.35 **
ADOLESCENCE AND ADULTHOOD							
Dagan et al. (2018) [61]	AAI	BDI, CES-D	Ds versus F	43	2881	0.09 (−0.03, 0.22)	Q(42) = 90.68 ***
			**E** versus F	38	2079	0.48 (0.30, 0.65)	Q(37) = 71.90 ***
			**E** versus Ds	37	1285	0.34 (0.19, 0.50)	Q(36) = 47.65
Dagan et al. (2020) [62]	AAI	BSI, SCL-90-R	Ds versus F	50	4376	−0.02 (−0.10, 0.05)	Q(49) = 68.09 *
			**E** versus F	42	3271	0.35 (0.19, 0.50)	Q(41) = 99.53 ***
			**E** versus Ds	41	2184	0.31 (0.15, 0.47)	Q(40) = 88.99 ***

Bolded letters represent the attachment classification groups associated with significantly more reported symptoms compared with the other group(s). *k* = Number of studies; N = Number of participants; AAI = Adult Attachment Interview; SSP = Strange Situation Procedure; BDI = Beck Depression Inventory (self-report); BSI = Brief Symptom Inventory (self-report); CBCL = Child Behavior Checklist (parent report); CES-D = Center for Epidemiologic Studies Depression Scale (self-report); SCL-90-R = Symptom Checklist-90-Revised (self-report); TRF = Teacher’s Report Form; A = Insecure-Avoidant; B = Secure; C = Insecure-Resistant; Ds = Insecure-Dismissing; E = Insecure-Preoccupied; F = Secure-Autonomous. * *p* < 0.05. ** *p* < 0.01. *** *p* < 0.001.
